# Symmetrical pH Electrochemical Cell Coupled to Constant Potential Coulometry for Improved Sensitivity and Precision: Part 1. Fundamental Considerations

**DOI:** 10.1021/acsmeasuresciau.5c00197

**Published:** 2026-02-10

**Authors:** Robin Nussbaum, Stéphane Jeanneret, Thomas Cherubini, Eric Bakker

**Affiliations:** Department of Inorganic and Analytical Chemistry, 27212 University of Geneva, Quai Ernest-Ansermet 30, 1211 Geneva, Switzerland

**Keywords:** constant potential coulometry, high sensitivity, pH glass electrode, seawater pH, symmetry

## Abstract

pH is a major variable in complex aquatic ecosystems, influencing biological activity, metal speciation and more. The method to routinely measure pH is potentiometric measurements with a glass electrode connected to a reference Ag/AgCl element in contact with the sample through a liquid junction. However, it has been largely replaced by optical pH assays in the field of seawater pH measurements because much better precisions could be achieved. Glass electrodes also suffer from bias such as liquid junction potential changes and temperature influence on the inner solution pH that generate inaccuracies. Moreover, the Nernstian relationship between observed potential and pH results in limited sensitivity. To overcome these limitations, an alternative readout called constant potential coulometry is implemented in the measurement system for increased sensitivity. A symmetrical pH cell is proposed in which two identical glass electrodes, separated by an open 3 M KCl channel, are measured against each other. One of them is kept in a NIST buffer while the other is used to measure the pH of the sample or the calibrant solution. Glass electrodes are evaluated at different temperatures in NIST buffers versus a classical reference electrode and versus each other in a symmetrical flow cell. The latter features open junctions that should improve the repeatability of the liquid junction potential. The determined signal repeatability in a single sample is as low as 0.3 mpH with a precision for consecutive samples of 0.001 pH, which is drastically improved over routinely available pH probes.

## Graphical abstract

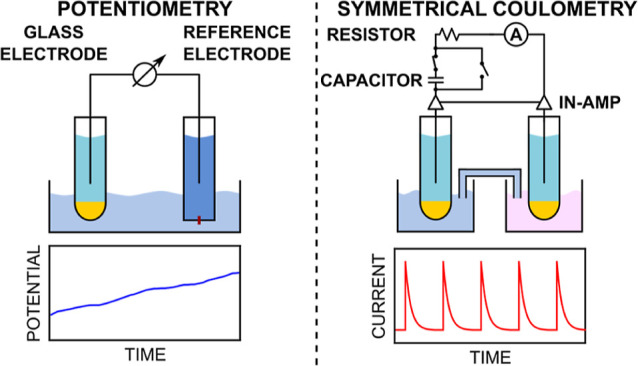

## Introduction

pH is considered as a master variable in aquatic systems as it affects a multitude of processes.^1^ It is therefore crucial to measure it both precisely and accurately.^2^ The pH glass electrode is certainly the most used ion-selective electrode as it is by far the most common pH measurement device. The first example was reported more than 100 years ago by Haber and Klemensiewicz.^3^ The phase-boundary potential at the sample-glass interface is known to depend on the hydrogen ion activity in the sample according to the Nernst equation^4^

1 $$E=E_{cell}^{{}^{\circ}}-\frac{RT\ln10}{F}pH=E_{cell}^{{}^{\circ}}-spH$$

where *E*_cell_^°^ is the standard potential of the cell, *R* the ideal gas constant, *T* the temperature, *F* the Faraday constant, *s* the Nernstian slope. Glass electrode is in most cases completed with an Ag/AgCl/3 M KCl reference electrode, typically combined inside the pH probe body and in contact with the solution trough a liquid junction.^5^ Highly concentrated KCl reduces the liquid junction potential variations with changing sample ionic strength and it therefore often used as salt bridge for reference electrodes.^6^ On the other hand, saturated KCl is not recommended since the temperature-dependent solubility would otherwise give rise to undesired potential changes at the reference element originating from temperature-dependent chloride activity. In oceanographic applications, seawater ingress into the salt bridge will give rise to potential drifts that are difficult to correct and predict. Traditional reference electrodes require maintenance and a defined overpressure for reliable operation. Potential drifts should be corrected by calibration, but standard NIST calibration buffers do not match the salinity of seawater, which is problematic. In the quest for improved precision and simplicity, potentiometric pH measurements have been largely abandoned by oceanographers for seawater measurements and replaced by indicator-based optical pH assays.^7^ This approach is based on the total pH scale (pH_T_) defined by oceanographers as the sum of the concentration of hydrogen and hydrogen sulfide ions.^2^ This definition is however not aligned with the fundamental definition of pH based on hydrogen ion activity.^8^ Ion activity is a more appropriate descriptor of the chemical driving force for aquatic chemical processes. Potentiometric measurements have a key advantage in that regard since they directly provide access to activity as parameter. We believe the sensing community could benefit from continued efforts to improve the performance of pH measurement systems based on glass electrodes, rather than moving away from them.

This work implements three different strategies to improve precision and stability of pH glass electrodes: (1) zero current potentiometry may be supplemented with a recently developed more sensitive method coined constant potential coulometry. (2) The nonideal variation of the inner solution pH with temperature is eliminated by measuring two identical pH probes against each other. (3) The instability of the liquid junction potential in contact with seawater is improved by implementing a renewable, open junction salt bridge.

Instead of zero-current potentiometry, an alternative readout method coined constant potential coulometry is implemented.^9^ In this protocol, an electronic capacitor, a resistor and a high impedance voltage follower are introduced in the measurement circuit between the electrochemical cell and the potentiostat. If the cell potential is held constant at a reference value during the measurement, any potential change at the sample-glass interphase induces an opposite potential change on the capacitor, resulting in a transient current.^10^ The latter is integrated to obtain the charge. This increases the sensitivity according to the following relationship between observed charge (*Q*) and pH^11^

2 $$Q=Cs\log\left(\frac{a_{i}\left(initial\right)}{a_{i}\left(final\right)}\right)=Cs\Delta pH$$

where *C* is the capacitance, *a*_*i*_ the activity of the hydrogen ion. However, the electronic circuit design from previous work, the CapaBoard, is not compatible with the high impedance of the second pH electrode in the symmetrical cell containing a pair of glass electrodes.^9^ An additional circuit, the Subtractor, was therefore designed and implemented here to solve this issue.

A routine combination pH glass electrode consists of the following cell

$$Ag\left|AgCl\right|a_{Cl^{-}}\left(3MKCl\right)∥sample\left|glass\right|a_{Cl^{-}}^{\prime },pH^{\prime }\left|AgCl^{\prime }\right|Ag^{\prime }\left(A\right)$$

where the apostrophe indicates the inner solution of the glass electrode and the double vertical bar a classical liquid junction. The composition of the inner solution of the latter cell is generally formulated so that the isopotential pH (pH_iso_, pH value at which the emf is invariant) is close to zero. The emf of cell A is given by eq 3.^12^

$$E={\left(E^{{}^{\circ}\prime }-{s\log a}_{{Cl}^{-}}^{\prime }+s{pH}^{\prime }\right)}_{glass}-spH+E_{j}-{\left(E^{{}^{\circ}}-s\log a_{{Cl}^{-}}\right)}_{ref}$$ 3

As many parameters depend on temperature, pH measurements require the standard solutions and the sample to be at the same temperature. This is relatively easily achieved in laboratories but very challenging when performing in situ measurements. The temperature influence on the pH measurement must therefore be known and characterized. Most commercial pH meters include a linear correction function with temperature but it was reported that all the processes in cell (A) do not vary in such manner with temperature.^12^ It is evident from eq 1 that *s* depends linearly on the temperature but the behavior of *E*_cell_^°^ with temperature is less trivial to determine. The majority of the parameters contained in *E*_cell_^°^ exhibit no or a linear temperature dependence but it is not the case for the pH′ of the majority of buffer-based inner solutions.^12^ One acceptable condition could be that δpH′/δ*T* would be equal to a constant. However, studies demonstrated that standard buffer solutions pH based on phosphate or carbonate do not exhibit a linear pH change with temperature.^13^ Buffers based on TES, HEPES and MOPS fulfilled this condition but failed to provide a pH_iso_ of 7.^14,15^ The group of Simon proposed back in 1964 a buffer recipe with a pH_iso_ of 7 measured against an Ag/AgCl-based reference electrode,^16,17^ giving a residual uncertainty of 10 mpH. This may be attractive for routine pH probes but not optimal for high precision aquatic measurements. Even with optimization of the inner solution, one problem remains for the glass electrode user: the nature of the inner solution is normally not disclosed by companies nor is its behavior with temperature.

For this reason, we propose to use a cell in which the emf between two identical glass electrodes in different compartments kept in electrical contact through a bridge. Differential measurements between two glass electrodes were already reported in the 1970s using multiple operational amplifiers combined with a ground electrode, with two example applications being environmental pH monitoring and pH titrations.^18,19^ More recently, this configuration was put forward to perform seawater pH measurement using an ionic liquid salt bridge.^20,21^ Unfortunately, this type of bridge was shown to introduce a larger accuracy error in seawater measurements than classical KCl salt bridge. On the other hand, open liquid junction designs with 3 M KCl were reported to provide very accurate pH measurement in dilute buffers or fresh waters and minimize errors in estuarine environments.^22–24^ In these works, 3 M KCl was injected from the bottom in a T-shaped junction and as it was denser than natural waters, the sample stayed on top, resulting in a well-defined junction. Moreover, in a classical pH measurement setup, the porous material of a restrained junction can be contaminated by the sample and clogged by precipitates including AgCl.^25^ The highly concentrated bridge can also contaminate the sample, influencing activity coefficients and therefore the pH.^25^

Given the above we propose a symmetrical pH measurement cell of the type

$${Ag}^{\prime }\left|{AgCl}^{\prime }\right|a_{Cl^{-}}^{\prime },pH^{\prime }\left|glass\right|sample\vdots 3MKCl\vdots ref\;buffer\left|glass\right|a_{Cl^{-}}^{\prime },pH^{\prime }\left|AgCl^{\prime }\right|Ag^{\prime },\left(B\right)$$

where vertical dots represent an open salt bridge. This system should nullify the effect of the pH change of the inner solution with temperature on the pH measurement, limit liquid junction potential change with ionic strength and minimize unexpected drifts to improve signal stability over time.

## Methods

### Materials and Instrumentation

Disodium hydrogen phosphate (Na_2_HPO_4_, >99%), potassium chloride (KCl, >99.5%), potassium dihydrogen phosphate (KH_2_PO_4_, >99%) were purchased from Sigma-Aldrich, Merck, Germany. Volumetric 1 M sodium hydroxide (NaOH) solution was purchased from Thermo Scientific. NIST/DIN buffer solutions of phthalate (pH = 4.008), phosphate (pH = 6.865) and borax (pH = 9.180) were purchased from Mettler Toledo, Switzerland. Ag/AgCl coated wires were purchased from Metrohm, Switzerland. Nafion tubing TT-060 was purchased from Perma Pure, USA. Idex Nonmetallic Inline Check Valves were purchased from Cole-Parmer, UK. The Ag/AgCl common reference elements electrodes are prepared by gluing a peek tube (10 cm outer diameter and 8 cm inner diameter) around an Ag/AgCl wire with the AgCl part left outside (Figure S1). A piece of Nafion tubing is sealed on one side by inserting a small piece of poly­(methyl methacrylate) (PMMA, Amsler & Frey AG, Switzerland). This small cap was then filled with 3 M KCl and fixed around the AgCl wire, creating a diffusion barrier for silver chloride complexes forming when AgCl is immersed in 3 M KCl. This prevents the dissolution of the AgCl layer and increases electrode lifetime. This assembly was then immersed in 3 M KCl. The pH glass electrodes were generously supplied by Metroglas AG, Switzerland. They are made of very robust glass with a long lifetime, similar to the ones sold to Idronaut, Italy, for implementation in submersible probes. Their impedance was determined to be around 50 MΩ.

The potentiometric measurements to study the inner solution of glass electrodes are done using a high impedance input 16-channel EMF monitor (Lawson Laboratories, Inc., Malvern, PA, USA) with a double junction Ag/AgCl/3 M KCl/3 M KCl reference electrode (Metrohm, Switzerland). When measuring only potentials in the flow cell, the two glass electrodes were plugged on two sample inputs, while the low-impedance common reference element was plugged on the reference channel of the instrument.

Capacitive readout is performed with an Autolab PGSTAT302N with homemade electronic circuits in between the potentiostat and the electrochemical cell. The main circuit, the Capaboard, was already presented in our previous work.^9^ The additional circuit, the Subtractor, is an add-on for symmetrical pH measurements. It is described in detail below. The current was recorded over at least 5 times the RC (resistance multiplied by capacitance) time constant to guarantee charging of the capacitor >99.3%. The temperature was controlled with a Dyneo DD-450F, Julabo, Germany, connected to an external thermostated cell. The solutions were pumped in the flow cell by a 4 channels peristatic pump (ISM935C, Ismatec, Switzerland) set at 2 mL/min.

### Flow Cell Design

The flow cell built for this work was machined from a PMMA piece and is depicted in Figure 1. A detailed scheme and a picture are presented in Figure S2 and S3 respectively. The cell includes two pH sensing compartments, containing one glass electrode each. The electrodes are held in place by silicone rubber (VMQ) O-rings. The sample glass electrode is in contact with the sample solution, while the reference glass electrode is kept in a NIST buffer with known behavior. The two pH sensing compartments are connected through a reference channel filled with 3 M KCl in which the low impedance common reference element is located. The reason for the presence of this third electrode is detailed in the following section. The sample and reference solutions are introduced from below the pH electrode into the sensing chambers to improve mixing. It is then guided down the elevation channel, whose purpose is to avoid 3 M KCl flowing back into the pH sensing compartment, and mixes with 3 M KCl in the T-shaped junction. The mixture then exits the flow cell through a check valve to prevent backflow caused by a small hydrostatic pressure difference in between exits. The reference solution follows an analogous path on its side of the flow cell.

**Figure 1:**
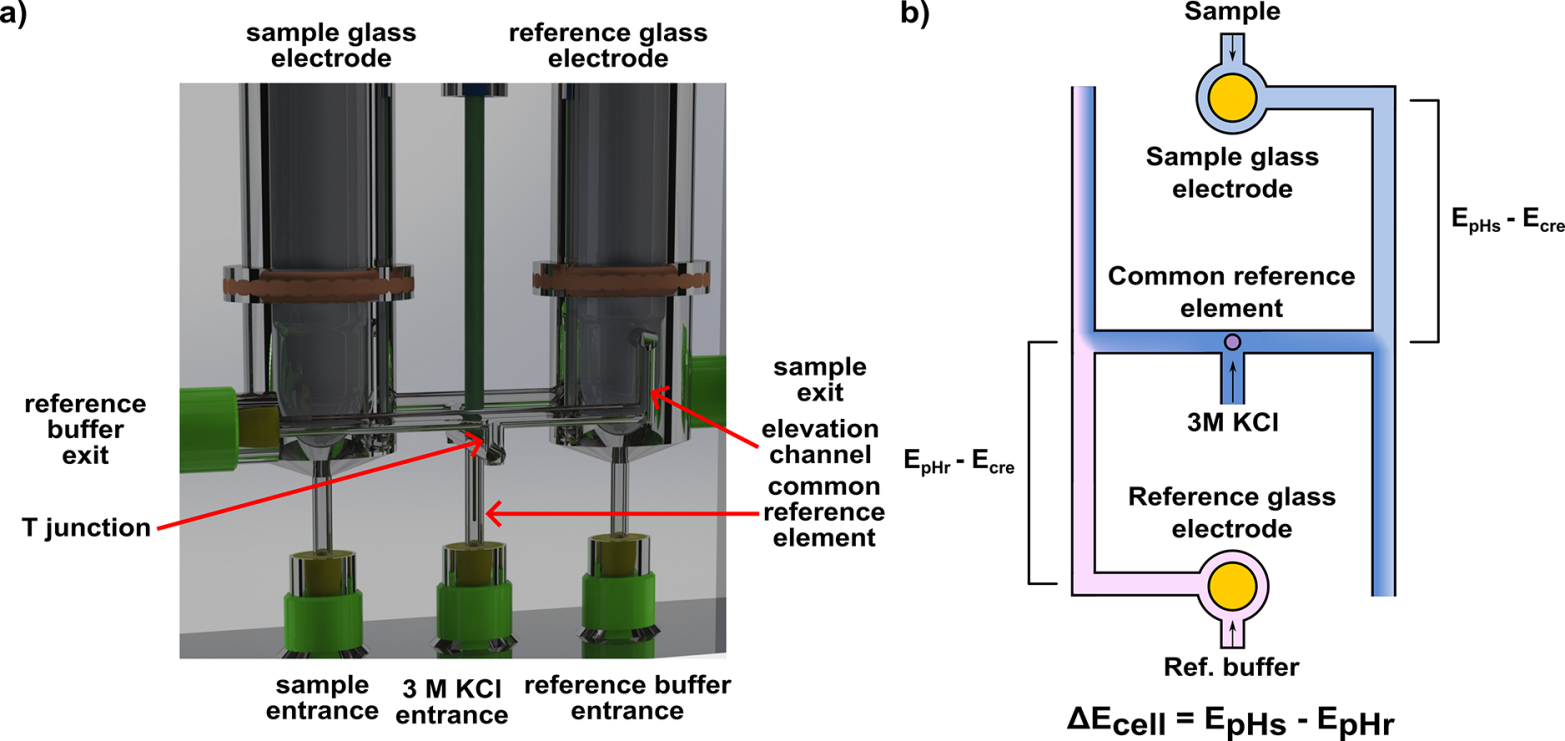
(a) Vertical image of the flow cell built for this work. The sample compartment is on the left, with the horizontal exit channel at the back. The reference compartment is on the right with the horizontal exit channel in the front. The 3 M KCl solution flows from the center and mixes with both solutions in T-shaped junctions. (b) Top-view schematics of the fluidic and electrical signals in the flow cell.

The T-shaped vertical open junction part is inspired by the work of Culberson, in which he described an open junction cell for pH sensing with 3 M KCl flowing from below.^22^ As 3 M KCl is denser than natural waters, both solutions are creating a stratified flow rather than mixing completely. When the flow is stopped for the measurement, the sample rests on the KCl solution, thereby improving the liquid junction potential reproducibility between measurements.

### Measurement Setup and pH Calculation Procedure

All experiments were performed in a temperature-controlled environment. For the beaker experiments, the electrodes were immersed in the buffer solution in a 50 mL closed thermostated cell. When performing experiments in the flow cell, the latter was immersed in a 250 mL thermostated cell and the solutions were placed in the main thermostated bath in closed glass Duran bottles. In this manner both the solutions and the sensors were at the same temperature. To perform a measurement, all 3 solutions (sample, reference buffer and 3 M KCl) were flown simultaneously at 2 mL/min for 3 min. This represented more than 3 times the volume of the input tubes and the flow cell, ensuring complete solution replacement. The 3 flows were stopped at the same time and after a rest period of 5 min, the signal was recorded.

For pH calibrations in the flow cell, a NIST phosphate buffer was prepared in house following the established composition.^5^ Aliquots of this solution were placed in different containers and the pH was adjusted with 1 M volumetric NaOH solution. The required addition volumes to achieve a given pH was determined with the Henderson–Hasselbalch equation. The solutions with altered pH were then pumped one after the other to cover the desired range of 0.05 pH units.

### Electronic Circuit and Coulometric Protocol

The Subtractor is capable of subtracting the voltage between two high impedance inputs. Pictures and electronic scheme can be found in Figures S4 and S5 respectively. It is intended to be placed between the electrochemical cell and the CapaBoard as depicted in Figure 2, top. The Subtractor contains an analog input stage (instrumentation amplifier, AD8220 chip) to measure the voltage difference between two points with a current down to 25 pA. It is powered with 9 V batteries. The dimension of the box is 17.5 × 8.2 × 4.9 cm and its weight is 475 g including batteries. This circuit requires the addition of a low impedance ground electrode in the flow cell, and an Ag/AgCl electrode coated by a Nafion membrane described above was used for this purpose. In fact, connecting a glass electrode to the low impedance ground input of the instrument would result in excessive noise to distinguish the current peaks. This electrode does not influence the readout of the electrochemical cell, as it is effectively canceled out when measuring both glass electrodes against each other. The Nafion layer improves the lifetime of the AgCl layer by eliminating the diffusion of silver chloro-complexes forming in highly concentrated KCl solutions which reduces the dissolution rate of the AgCl layer. Moreover, using an Ag/AgCl wire in 3 M KCl allows for a stable reference potential that allows one to probe each glass electrode individually when needed. As in previous work, the connection of the electrodes to the potentiostat results in an opposite sign of the potential response than traditionally reported.

**Figure 2:**
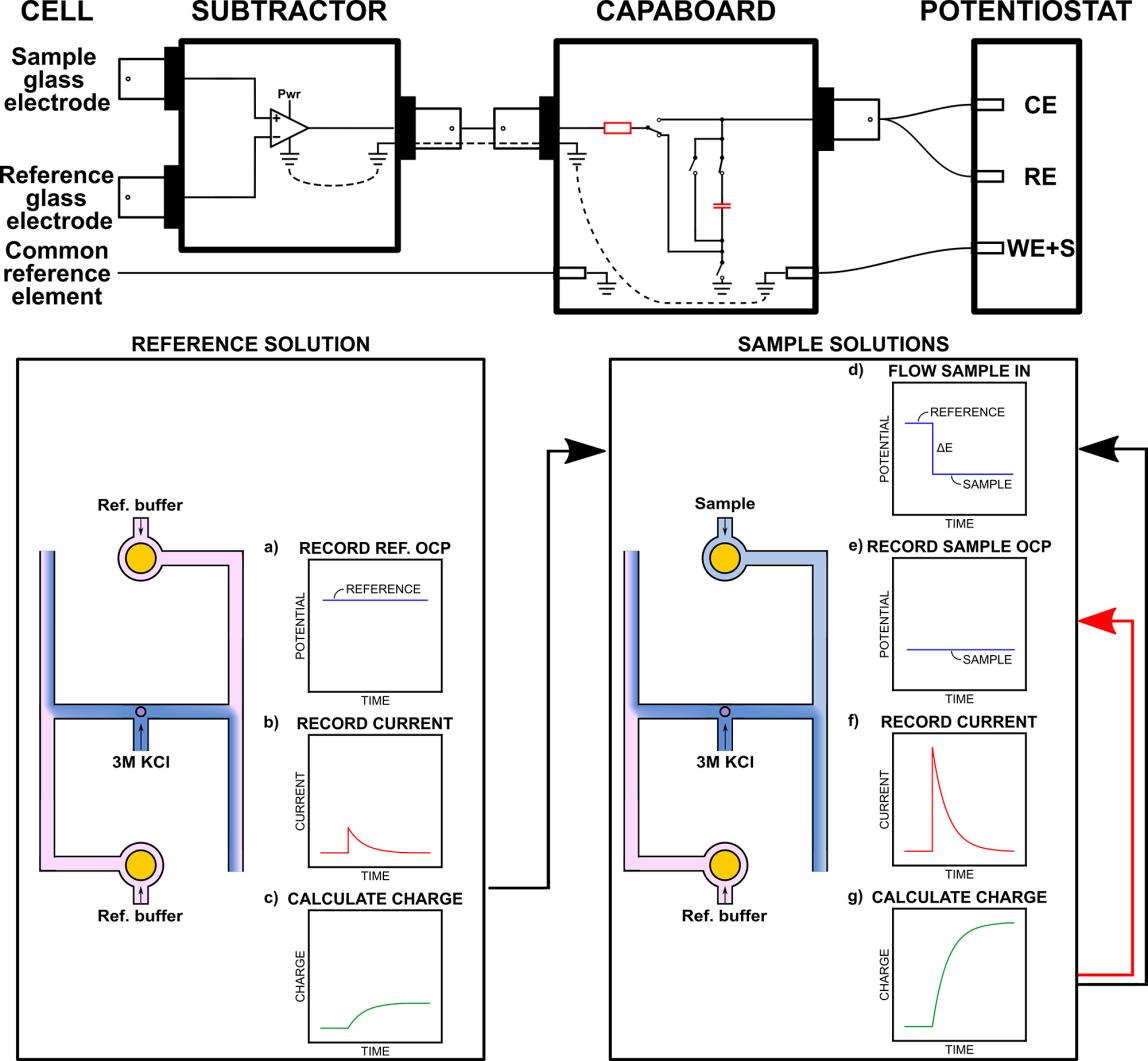
Top: Experimental setup for coulometric readout in the symmetrical cell. The capacitor and resistor producing the current are highlighted in red. The dotted lines are to help the reader understand the current path through the common ground of the system during the coulometric measurement. Bottom: Coulometric procedure in the flow cell. (a) The OCP_ref_ is recorded with both pH electrodes immersed in the reference buffer. (b,c) The instrumental charge offset is then measured. (d) The sample is flown in the flow cell. (e–g) The OCP and charge are recorded in the sample solution. The latter steps are repeated 3 times in the same solution.

The steps of coulometric protocol are shown in Figure 2, bottom. The first is to record the OCP_ref_ of the circuit with both glass electrodes placed in the same buffer solution (Figure 2a). This should, in an ideal case, give a 0 mV reading. The reference potential is then enforced to the same solution to measure any residual current and charge caused by the applied potential, which is never exactly OCP_ref_ (Figure 2b,c). This charge, referred to as instrumental charge offset, is then subtracted to all sample measurements. The sample is then pumped through the flow cell (Figure 2d). After a settling period of 5 min, the OCP of the sample solution is recorded for potentiometric readout (Figure 2e). Then, the reference OCP is enforced by the potentiostat and, as there is a potential mismatch in the circuit, the capacitor charges with a transient current and the charge is obtained by integration (Figure 2f,g). The capacitor is then be short-circuited electronically. Steps (e) to (g) are repeated 3 times for each sample for signal repeatability assessment (red arrow). Then, a new sample can be flown through the flow cell (black arrow) and the procedure can be carried on like this as long as required. Signal repeatability refers to the standard deviation measurements carried out in the very same sample. In this work, three potentiometric and coulometric measurements were performed for each sample. The average signal repeatability refers to the average of the signal repeatabilities for different samples. Precision is defined as the standard deviation of the pH values obtained for different samples of which the composition is assumed to remain constant. Average precision is the average of the calculated precisions for each sample. More details about the functioning of the Capaboard (characteristics, electronic schemes and detailed electronic workflow of the coulometric protocol) may be found in our previous work.^9^

## Results and Discussion

The behavior of a pair of glass electrodes was investigated at 25 °C versus a double junction Ag/AgCl/3 M KCl/3 M KCl reference electrode by immersing them successively in NIST phthalate, phosphate and borax. They both exhibited a slope of −58.6 mV/pH (99% of Nernstian behavior) and their zero-point pH (pH_0_) was 6.96, which is close to the typical pH_0_ of 7. Both these electrodes were introduced in the symmetrical flow cell to evaluate the behavior of the system at a fixed temperature of 25 °C. The reference compartment was filled with NIST phosphate and the sample compartment was filled successively with NIST phthalate, phosphate and borax (Figure 3). The slope was close to Nernstian (−58.7 mV, 99% of expected value), indicating that the glass electrode in the reference compartment can be treated as a reference electrode. The pH_0_ was 6.86 and lies in the accuracy range of the phosphate buffer stated by the manufacturer (6.865 ± 0.015) and the potential when both electrodes are in the same solution was 0.7 mV, which is acceptable for a symmetrical system.

**Figure 3:**
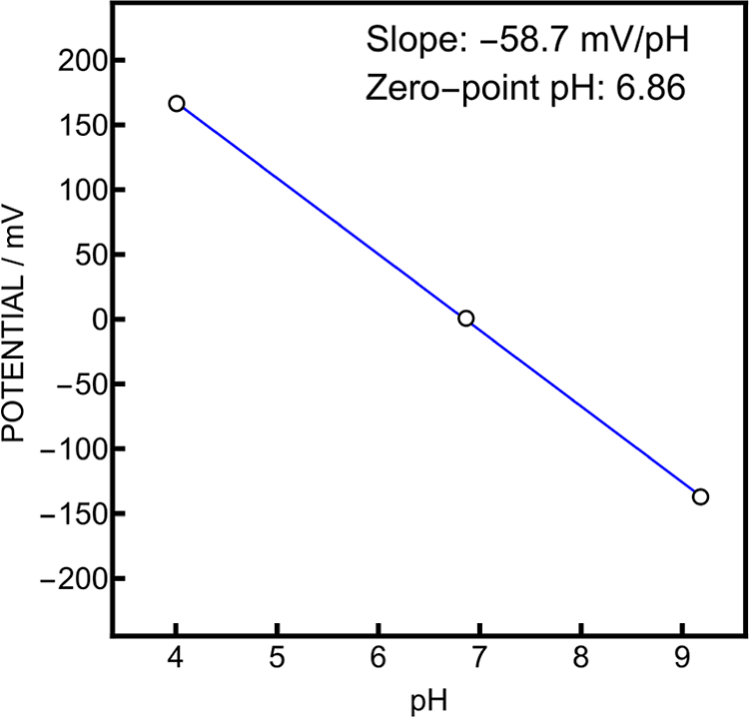
pH calibration in the symmetrical flow cell with NIST phosphate in the reference compartment.

Now that the behavior of the cell is assessed at 25 °C, the performance of the system with temperature can be evaluated. From the introduction it is expected that the inner solution pH, and hence the pH_0_, changes with temperature. To test this experimentally, the behavior of two other glass electrodes was evaluated versus a commercial Ag/AgCl/3 M KCl/3 M KCl reference electrode with changing temperature. The 3 electrodes were immersed into NIST borax buffer contained in a closed thermostated cell. The temperature was lowered from 25 to 5 °C with steps of 5 °C. This temperature range was chosen as it covers the temperatures that are expected in the field evaluation described in Part 2 of this work.^26^ The OCP was recorded for each temperature and the ramp was repeated 3 times, with each temperature ramp being treated individually (Figure S6a). Then, the same experiment was repeated in NIST phosphate buffer (Figure S6b). The OCP values at 25 °C in both NIST borax and NIST phosphate were used to determine the slope and the pH_0_. The slopes at lower temperatures were calculated from this experimentally determined slope and the pH_0_ was assumed to be independent of temperature. This way, any deviation from an ideal behavior with changing temperature would refute the assumption that the pH_0_ is constant. The ideal behavior was obtained by fitting the reported pH values from the manufacturer with a previously published model at the experimental temperatures.^27^ The comparison between ideality and experimental results for the two glass electrodes are presented in Figure 4a. As both curves deviate from ideality by up to 20 mpH, it is clear that the pH_0_ does indeed change with temperature. Surprisingly, the two glass electrodes do not show exactly the same behavior in the two buffers. When subtracting the response of the two glass electrodes, the results for borax and phosphate buffers become closer, suggesting that their previously observed difference originates in the liquid junction potential that is different for both buffers (Figure 4b). In fact, the species and concentrations in the sample are quite different between NIST phosphate and borax.^27^ It was reported that the uncertainty of the liquid junction potential between these two buffers was between 0.005 and 0.010 pH units.^28^

**Figure 4:**
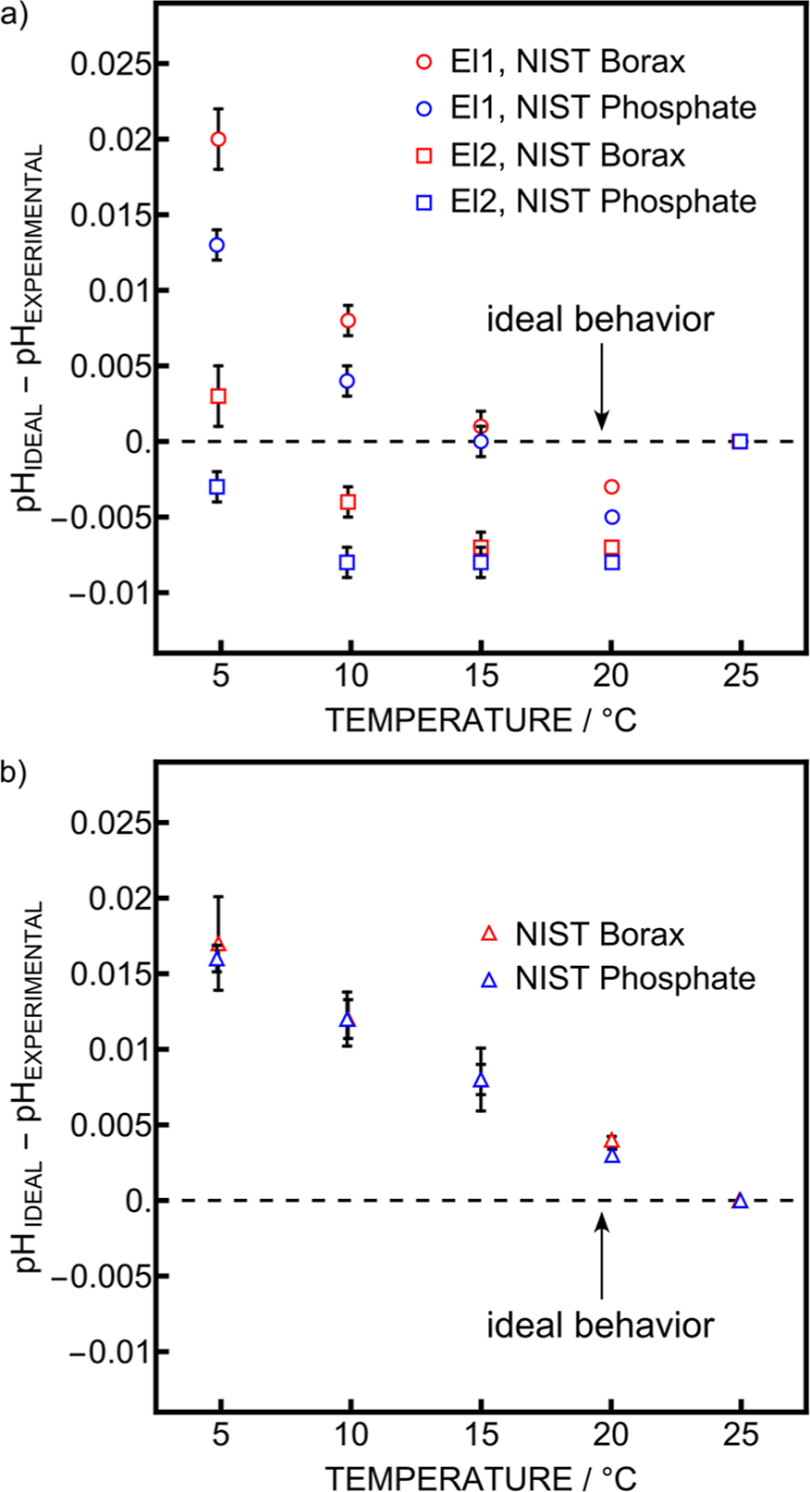
(a) Comparison between ideal and experimental pH in two NIST buffers for two glass electrodes measured again a 3 M KCl reference electrode. Error bars represent standard deviations between 3 replicates at a given temperature. (b) Comparison between ideal and experimental pH in two NIST buffers for two glass electrodes measured against each other. The error bars represent the combination of the standard deviation of both electrodes at each temperature.

One hypothesis of this work is that two identical glass electrodes should behave more similarly with changing temperature than a glass electrode measured against a reference electrode. The symmetrical flow cell was used to perform similar temperature ramps than above. Since the main application of this work is pH sensing in natural waters, and calcium phosphate salts are known to have low solubility, the buffer in the reference compartment was chosen to be NIST borax for this application. It is suitable because it is used as primary standard and its pH is close to that of natural waters. The temperature was again lowered from 25 to 5 °C with steps of 5 °C and NIST borax buffer was flown in the sample compartment 3 times at each temperature (Figure S7). The potential drift present in Figure S7a is nicely canceled out when measuring both glass electrodes against each other (Figure S7b). The experiment was then repeated with NIST phosphate buffer (Figure S8). For each temperature, both buffers were used to build calibration curves and the pH_0_ was determined. In an ideal case, it should follow the pH of NIST borax buffer because the potential should be 0 mV when the same buffer is present in both compartments. However, the OCP recorded at 25 °C was not 0 mV but was 2.0 mV, indicating an asymmetry. This value was therefore subtracted to all other OCPs when determining the pH_0_ values. Moreover, the gradual increase of the deviation between the 2 electrodes with decreasing temperature observed in Figure S7b also points out that both electrodes are not identical. The difference between experimental data and theoretical predictions of pH_0_ are shown in Figure 5. One notices that the deviation from ideality diminish to less than 5 mpH compared to an asymmetrical system (up to 20 mpH) but does not cancel out fully. This points out that glass electrodes from the same batch do not all behave identically. As both electrodes contain the same inner solution, the same inner AgCl element and are sealed, this deviation is expected to originate from the glass membranes itself. In fact, it has been known since Cremer’s work in 1906 that different sides of a glass membrane do not behave exactly the same.^29^ This was referred to as asymmetry potential and may be due to multiple factors such as changes of the hydrogen ion exchange capacity, modification of the glass during blowing in a flame or different strains on the glass bulbs.^27,30,31^ If such difference can be observed within the same electrode, one should assume that differences do occur between glass electrodes from the same batch. Improving consistency between glass electrodes would be a worthwhile effort but outside the scope of this work. A limitation of this approach is that the accuracy of the pH measurement largely depends on the accuracy of the reference buffer used. In the case of this work, it is stated by the manufacturer as 0.015 pH unit. The use of primary standards whose uncertainty is typically around 0.003 pH unit will improve accuracy at the cost of affordability and availability. Moreover, the systematic uncertainty resulting from liquid potential changes when moving from NIST buffers to seawater is not eliminated with this approach. A characterization of the liquid junction potential at variable salinities^32^ was however outside of the scope of this work.

**Figure 5:**
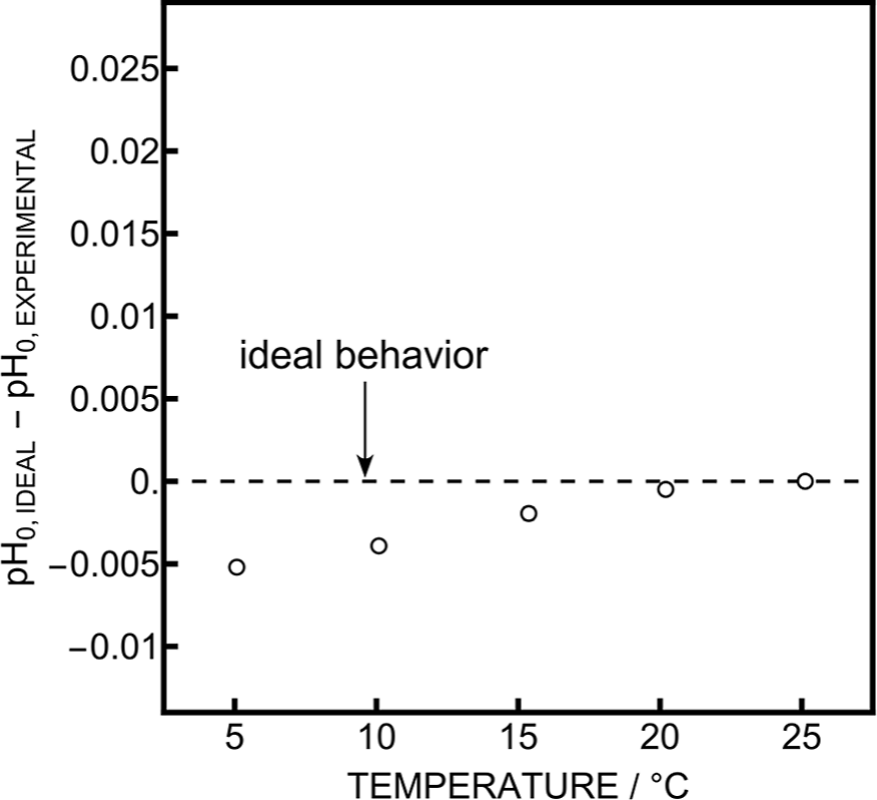
Comparison between ideal and experimental pH_0_ in the symmetrical flow cell at different temperatures.

The behavior of the combination of the CapaBoard and Subtractor was directly tested with glass electrodes in the symmetrical flow cell with NIST borax in the pH reference compartment. The measurement reproducibility and precision were evaluated by performing a pH calibration with 0.01 pH steps over a 0.05 pH range at a constant temperature of 25 °C. The reference potential was first recorded in a “NIST” phosphate buffer solution (pH = 6.865; prepared in house) and phosphate buffer solutions with altered pH were then measured successively. Each solution was replaced and measured 3 times with potentiometric and coulometric signals, with 3 consecutive recordings for each sample replacement. The transient current spikes obtained for the first pumping of each solution are given in Figure 6a with the corresponding integrated charge shown in Figure 6b. Potentiometric calibration gave a response slope of 61.9 mV with an average signal repeatability of 195 μpH (Figure 6d). An opposite response is expected compared to Nernstian response because the glass electrodes are connected to the reference channel of the instrument. The observed seemingly excellent repeatability is not very meaningful since the value is lower than the instrumental resolution of 500 μpH, see discussion in earlier work.^9^ The coulometric calibration exhibited a higher slope of −30.8 μC, corresponding to −63.9 mV (Figure 6c). The average signal repeatability was in this case 354 μpH, which is similar to the results obtained in previous work^9^ and again better than the potentiometric resolution of the instrument. For both readouts, the precision between 3 samples of identical pH was 1 mpH. This precision approaches that of indicator-based pH assays and therefore attractive for seawater pH measurements,^33^ although further improvements should be necessary to monitor ocean acidification processes on a long time scale as its rate is just −2 mpH y^–1^.^34,35^ The difference in slope between the two readouts likely originates from the electronic components of the instrument. It seems therefore advisible to calibrate both methods individually for accurate measurements.

**Figure 6:**
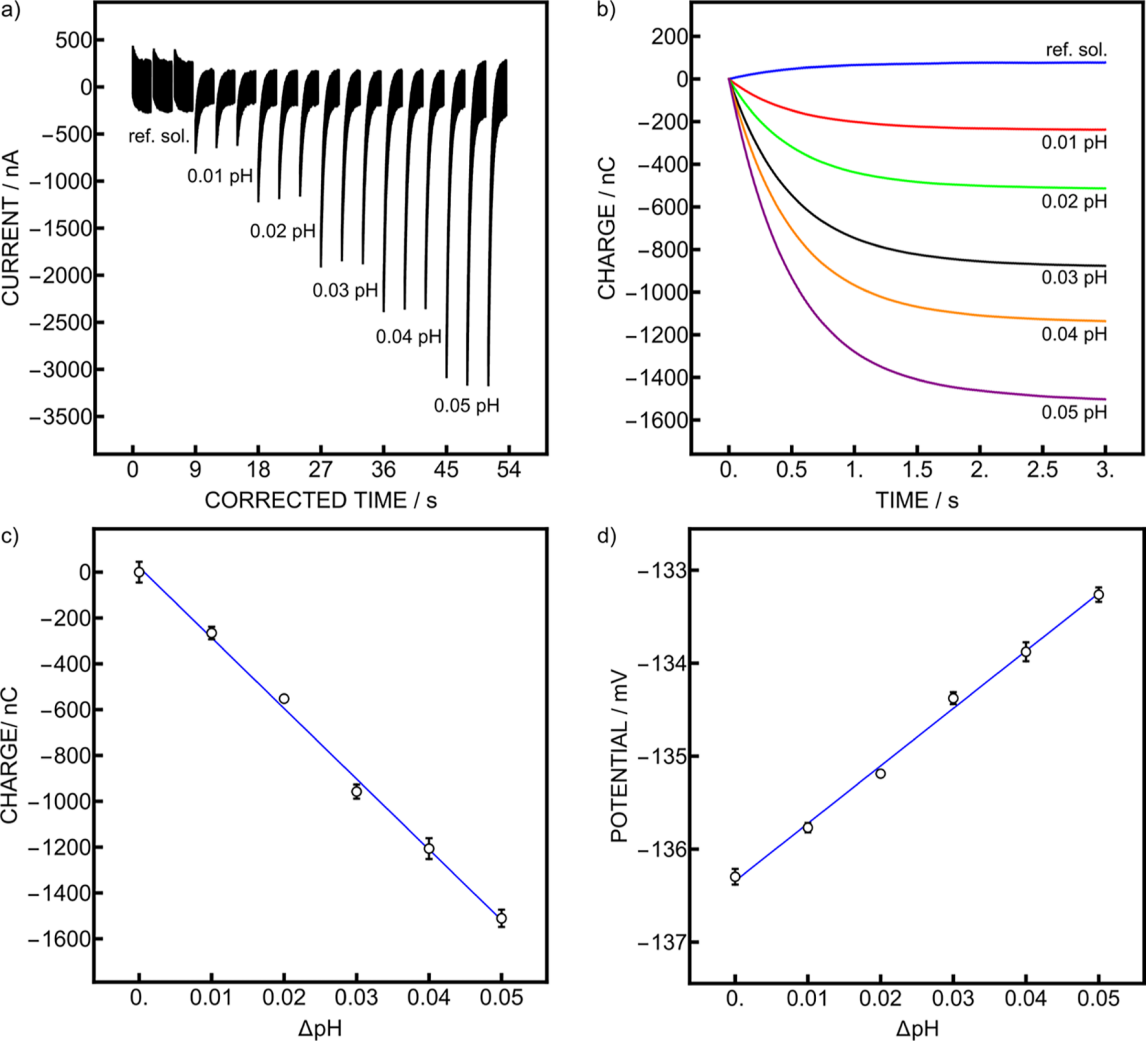
(a) Current traces for one flow of each solution. (b) An example of charge obtained from integration of current for each solution pH. (c) Coulometric and (d) potentiometric calibration curves obtained from the whole experiment. Error bars represent standard deviations of 3 consecutive samples for each pH.

## Conclusions

This work demonstrates the use to chemical symmetry to reduce undesired errors during pH sensing with temperature variations. This approach allows one to minimize the influence of inner solution pH changes as a function of temperature to less than 5 mpH, which otherwise introduces an uncertainty of about 20 mpH. The residual uncertainty is explained with structural differences of the glass membranes of different electrodes, even from the same production batch. It would be welcome for this characteristic to be further improved by pH glass electrode manufacturers. Constant potential coulometry circuits were adapted from previous work to allow for constant potential coulometric measurements with two glass electrodes. The signal reproducibility was comparable to previous work and higher than the potentiometric instrumental resolution. Precision as high as 1 mpH was observed for 10 mpH changes, which is much better than with routine pH probes and suitable for seawater pH monitoring. Further characterization of this system should include a study of salinity effect on the liquid junction potential and comparison with optical pH measurements to assess the systematic uncertainties and accuracy of the proposed method.

This work aimed to help transition laboratory experiments at controlled temperature to allow for high precision measurements of pH in situ, under variable field conditions. For this reason, the flow cell was designed and built to be directly implemented as the sensing head of a submersible electrochemical probe containing dedicated readout instrumentation developed in house to perform potentiometric and coulometric measurements. The results of the deployment of this submersible probe in the vertically stratified Krka River Estuary in Croatia are reported in part 2 of this work.^26^ There, signal stability is evaluated in the seawater layer and vertical profiling is conducted to measure pH variations associated with salinity changes. Signal drift was also compensated by in situ recalibration with a matrix-matched buffer.

## Supplementary Material



## Notes

Scheme of the common reference element electrode, building scheme and picture of the flow cell, pictures and electronic scheme of the Subtractor, potential traces during the temperature beaker and flow cell experiments can be found in the Supporting Information. part1SI.pdf (PDF)

## References

[1] Stumm, W. ; Morgan, J. J. Aquatic Chemistry, 2nd ed.; John Wiley & Sons: New York, 1981.

[2] Marion G. M., Millero F. J., Camões M. F., Spitzer P., Feistel R., Chen C.-T. A. (2011). pH of Seawater. Mar. Chem..

[3] Marczewska B., Marczewski K. (2010). First Glass Electrode and Its Creators F. Haber and
Z. Klemensiewicz – On 100th Anniversary. Z. Phys. Chem..

[4] Morf W. E. (1979). A Unified
Approach to Glass Electrode Theory. Talanta.

[5] Buck R. P., Rondinini S., Covington A. K., Baucke F. G. K., Brett C. M. A., Camoes M. F., Milton M. J. T., Mussini T., Naumann R., Pratt K. W., Spitzer P., Wilson G. S. (2002). Measurement of pH.
Definition, Standards, and Procedures (IUPAC Recommendations 2002). Pure Appl. Chem..

[6] Troudt B. K., Rousseau C. R., Dong X. I. N., Anderson E. L., Bühlmann P. (2022). Recent Progress
in the Development of Improved Reference Electrodes for Electrochemistry. Anal. Sci..

[7] Clayton T. D., Byrne R. H. (1993). Spectrophotometric Seawater pH Measurements: Total
Hydrogen Ion Concentration Scale Calibration of *m*-Cresol Purple and at-Sea Results. Deep Sea
Res., Part I.

[8] Sörensen, Søren P. L., Enzymstudien I. I. (1909). Über die Messung und die Bedeutung der Wasserstoffionenkonzentration
bei enzymatischen Prozessen. Biochem. Z..

[9] Nussbaum R., Jeanneret S., Bakker E. (2024). Increasing the Sensitivity
of pH
Glass Electrodes with Constant Potential Coulometry at Zero Current. Anal. Chem..

[10] Kraikaew P., Jeanneret S., Soda Y., Cherubini T., Bakker E. (2020). Ultrasensitive Seawater pH Measurement by Capacitive
Readout of Potentiometric Sensors. ACS Sens..

[11] Jarolímová Z., Han T., Mattinen U., Bobacka J., Bakker E. (2018). Capacitive Model for
Coulometric Readout of Ion-Selective Electrodes. Anal. Chem..

[12] Midgley D. (1987). Temperature
Compensation in Potentiometry: Isopotentials of pH Glass Electrodes
and Reference Electrodes. Part I. Theory. Analyst.

[13] Perrin, D. D. ; Dempsey, B. Buffers for pH and Metal Ion Control; Springer Netherlands: Dordrecht, 1979.

[14] Bates R. G., Vega C. A., White D. R. (1978). Standards for pH
Measurements in
Isotonic Saline Media of Ionic Strength I= 0.16. Anal. Chem..

[15] Sankar M., Bates R. G. (1978). Buffers for the Physiological pH Range: Thermodynamic
Constants of 3-(N-Morpholino) Propanesulfonic Acid from 5 to 50. Degree.
C. Anal. Chem..

[16] Wegmann D., Simon W. (1964). Glaselektroden-Messkette Mit Isothermenschnittpunkt Bei pH= 7, 0
Zur pH-Messung Unter Extremen Bedingungen [1]. Helv. Chim. Acta.

[17] Clerc J. T., Štefanac Z., Simon W. (1965). Temperaturabhängigkeit
Des
Potentials von Glaselektroden-Messketten. Einfaches Hochtemperatur-Ableitsystem. Helv. Chim. Acta.

[18] Wilde P., Rodgers P. W. (1970). Electrochemical Meter for Activity
Measurements in
Natural Environments. Rev. Sci. Instrum..

[19] Light T. S., Swartz J. L. (1977). Null-Point Control
of Acidity with a Differential pH
Electrode System. Anal. Chem..

[20] Lainela S., Leito I., Heering A., Capitaine G., Anes B., Camões F., Stoica D. (2021). Toward Unified pH of
Saline Solutions. Water.

[21] Heering A., Stoica D., Camões F., Anes B., Nagy D., Nagyné Szilágyi Z., Quendera R., Ribeiro L., Bastkowski F., Born R., Nerut J., Saame J., Lainela S., Liv L., Uysal E., Roziková M., Vičarová M., Snedden A., Deleebeeck L., Radtke V., Krossing I., Leito I. (2020). Symmetric Potentiometric
Cells for the Measurement of Unified pH Values. Symmetry.

[22] Culberson, C. H. Direct Potentiometry. In Marine Electrochemistry; Whitfield, M. , Jagner, D. , Eds.; John Wiley and Sons: Chichester, 1981; pp 187–201.

[23] Whitfield M., Butler R. A., Covington A. K. (1985). The Determination
of pH in Estuarine
Waters. I: Definition of pH Scales and the Selection of Buffers. Oceanol. Acta.

[24] Butler R. A., Covington A. K., Whitfield M. (1985). The Determination of pH in Estuarine
Waters. II: Practical Considerations. Oceanol.
Acta.

[25] Covington A. K., Whalley P. D., Davison W. (1985). Recommendations for the Determination
of pH in Low Ionic Strength Fresh Waters. Pure
Appl. Chem..

[26] Nussbaum, R. ; Jeanneret, S. ; Cherubini, T. ; Tercier-Waeber, M.-L. ; Omanović, D. ; Bakker, E. Symmetrical pH Electrochemical Cell Coupled to Constant Potential Coulometry for Improved Sensitivity and Precision: Part 2. Submersible Probe for In Situ Measurements. ACS Meas. Sci. Au 2026. 10.1021/acsmeasuresciau.5c00198 PMC13087930 42007057

[27] Bates, R. G. Determination of PH: Theory and Practice; Wiley, 1973.

[28] Covinton A.
K., Rebelo M. J. F. (1987). Determination
of pH Values over the Temperature Range
– 60°C for Some Operational Reference Standard Solutins
and Values of the Conventional Residual Liquid-Junction Potentials. Anal. Chim. Acta.

[29] Cremer M. (1906). Über
Die Ursache Der Elektromotorischen Eigenschaften Der Gewebe, Zugleich
Ein Beitrag Zur Lehre von Den Polyphasischen Elektrolytketten. Z. Biol..

[30] Yoshimura H. (1937). Studies on
the Nature of the Glass Electrode Potential. III. On the Cause of
the Asymmetry Potential of the Glass Electrode. Bull. Chem. Soc. Jpn..

[31] Kratz, L. Die Glaselektrode und Ihre Anwendungen; Verlag Dr; Dietrich Steinkopff: Frankfurt am Main, 1950.

[32] Bagg J. (1993). Temperature
and Salinity Dependence of Seawater-KCl Junction Potentials. Mar. Chem..

[33] Dickson, A. G. ; Sabine, C. L. ; Christian, J. R. ; Bargeron, C. P. Guide to Best Practices for Ocean CO_2_ Measurements; PICES special publication; North Pacific Marine Science Organization: Sidney, BC, 2007.

[34] Bates N. R., Best M. H. P., Neely K., Garley R., Dickson A. G., Johnson R. J. (2012). Detecting Anthropogenic Carbon Dioxide
Uptake and Ocean
Acidification in the North Atlantic Ocean. Biogeosciences.

[35] González-Dávila M., Santana-Casiano J. M. (2023). Long-Term Trends of pH and Inorganic Carbon in the
Eastern North Atlantic: The ESTOC Site. Front.
Mar. Sci..

