# Supplementary material for: Symmetrical pH Electrochemical Cell Coupled to Constant Potential Coulometry for Improved Sensitivity and Precision: Part 1. Fundamental Considerations
Source: ACS Meas Sci Au. 2026 Feb 10;6(2):488–96. doi: 10.1021/acsmeasuresciau.5c00197 (PMC13087951; doi:10.1021/acsmeasuresciau.5c00197)
Supplement: Supplementary file 1 [file tg5c00197_si_001.pdf]

Supplementary Information for:

Symmetrical pH Electrochemical Cell Coupled to Constant Potential Coulometry for Improved Sensitivity and Precision: Part 1. Fundamental Considerations

Robin Nussbaum, Stéphane Jeanneret, Thomas Cherubini, Eric Bakker*

Department of Inorganic and Analytical Chemistry, University of Geneva, Quai Ernest-Ansermet 30, 1211 Geneva, Switzerland.

*Eric.Bakker@unige.ch

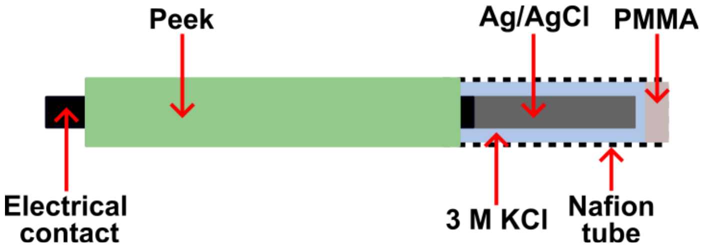

Figure S1. Scheme of the common reference element used in this work.

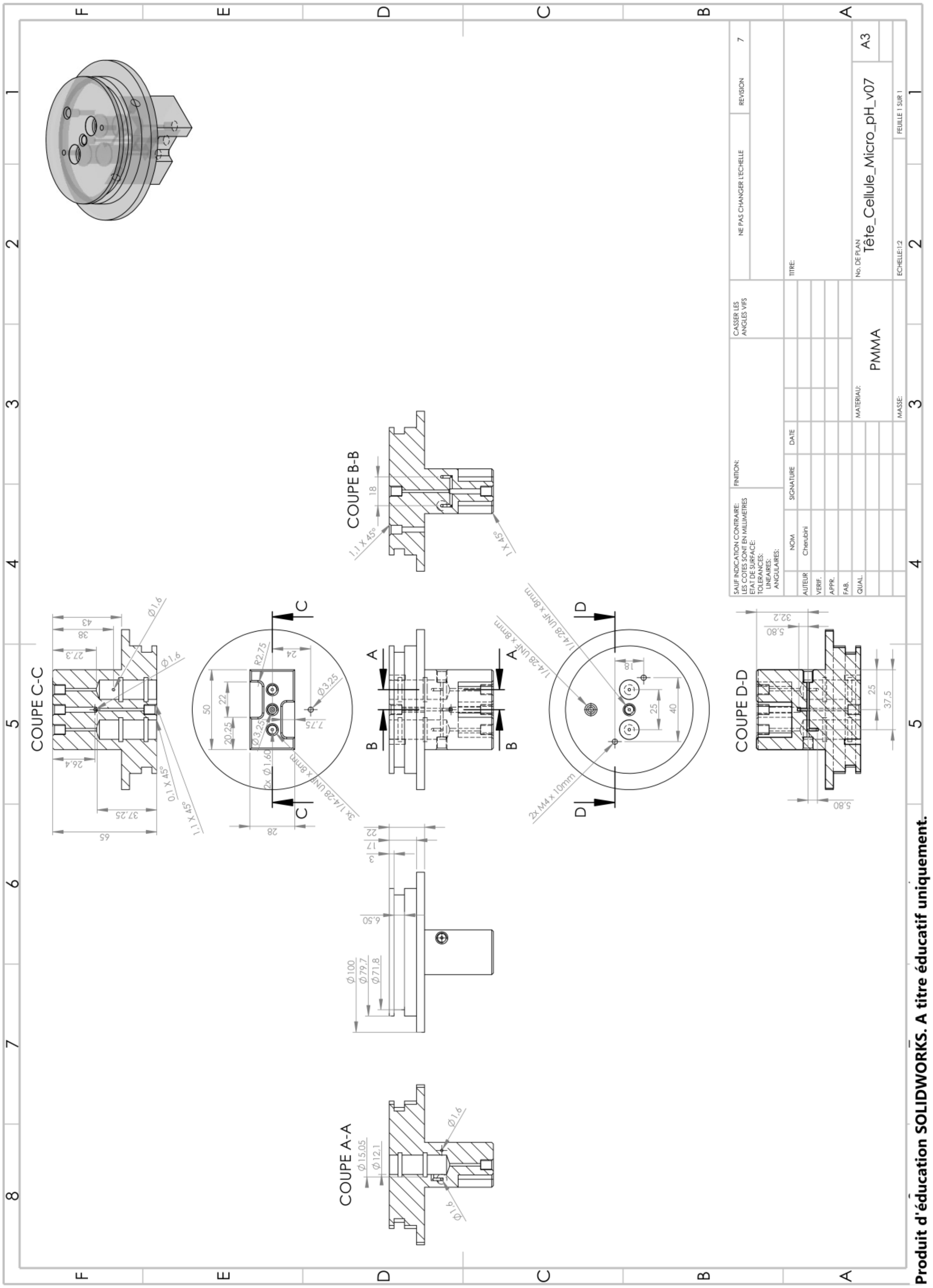

Figure S2. Detailed schemes of the flow cell.

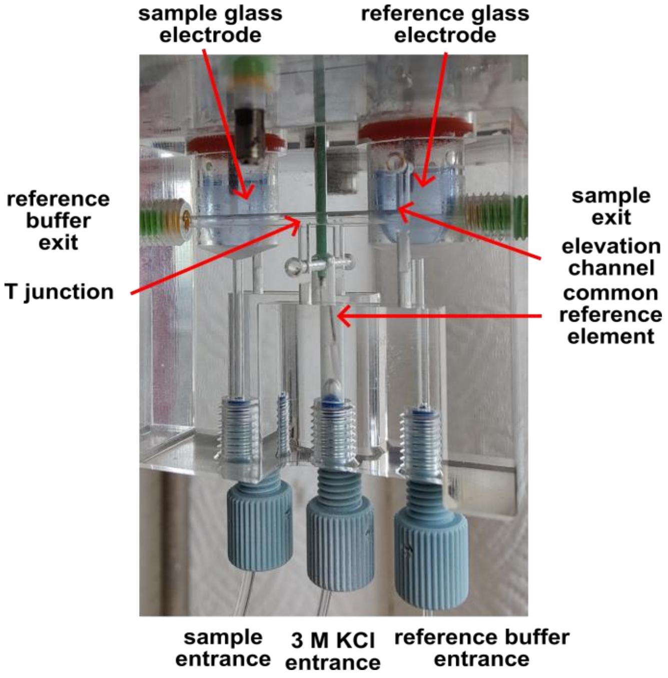

Figure S3. Picture of the flow cell.

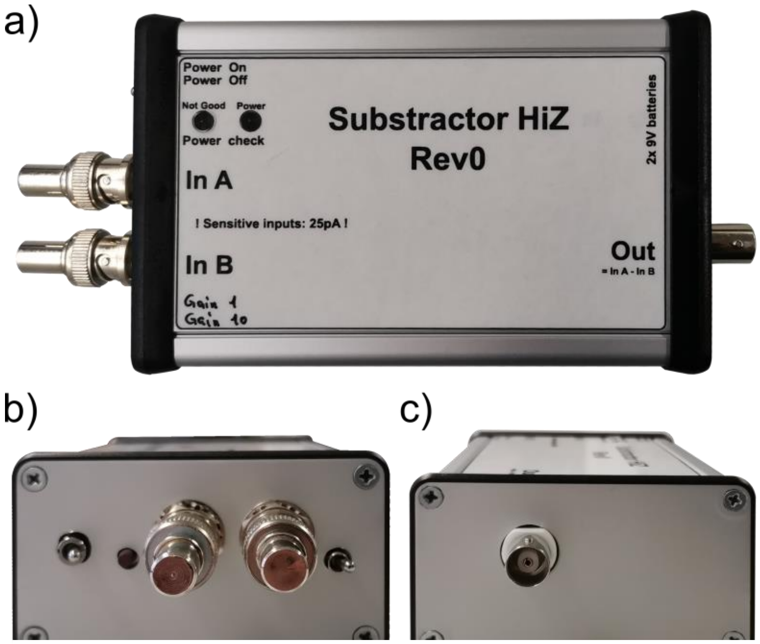

Figure S4. a) top, b) left, c) right views of the Subtractor box.

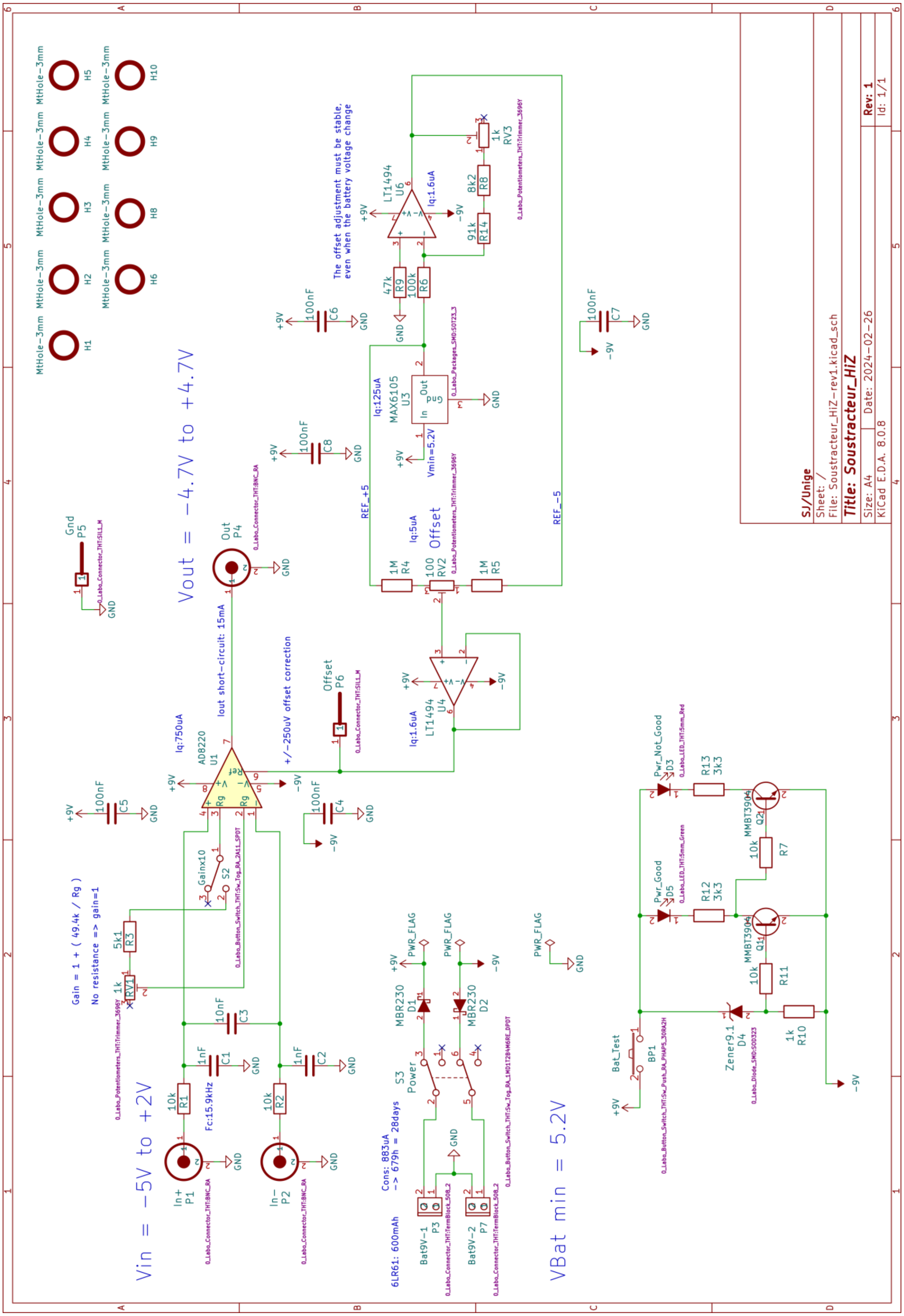

Figure S5. Electronic scheme of the Subtractor.

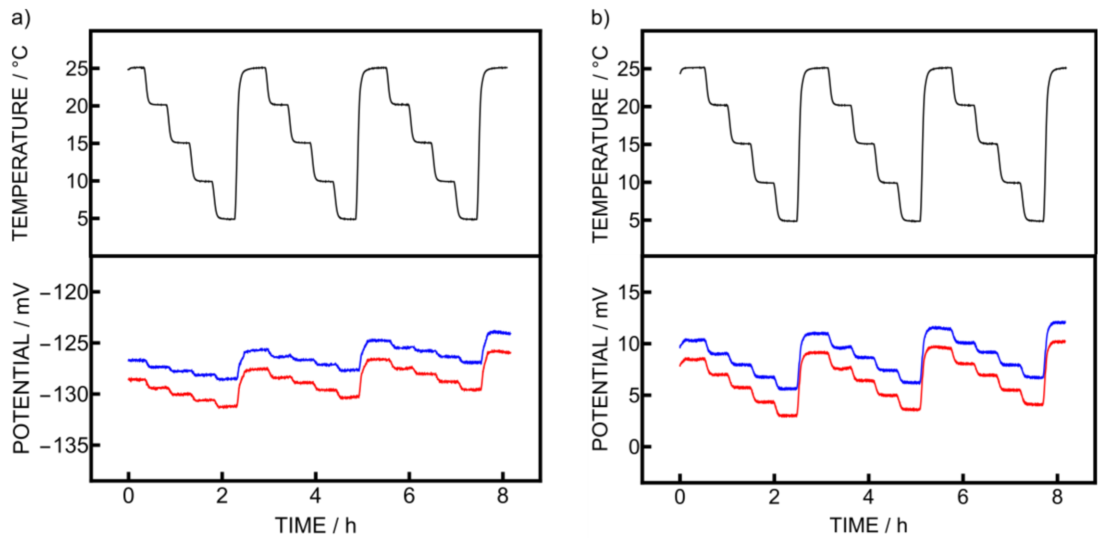

Figure S6. Potential trace of sample (blue) and reference (red) glass electrode recorded in a thermostatted cell against an Ag/AgCl/3 M KCl/3 M KCl reference electrode in a) NIST borax and b) NIST phosphate buffers.

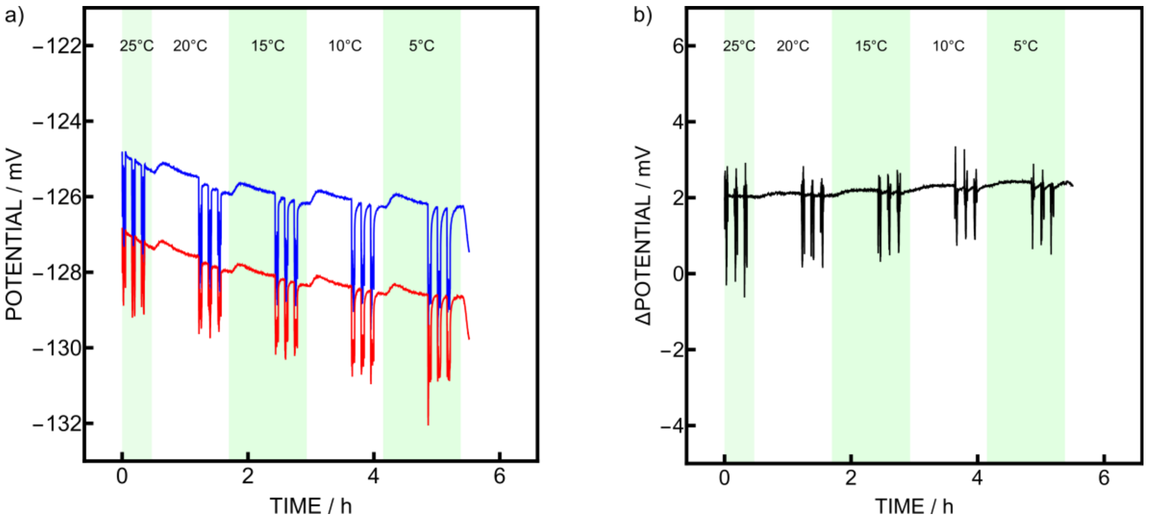

Figure S7. a) Potential trace of sample (blue) and reference (red) pH glass electrodes against common reference element in the symmetrical flow cell with NIST borax buffer in both compartments at different temperatures. b) Corresponding potentiometric trace of the sample glass electrode measured against the reference glass electrode.

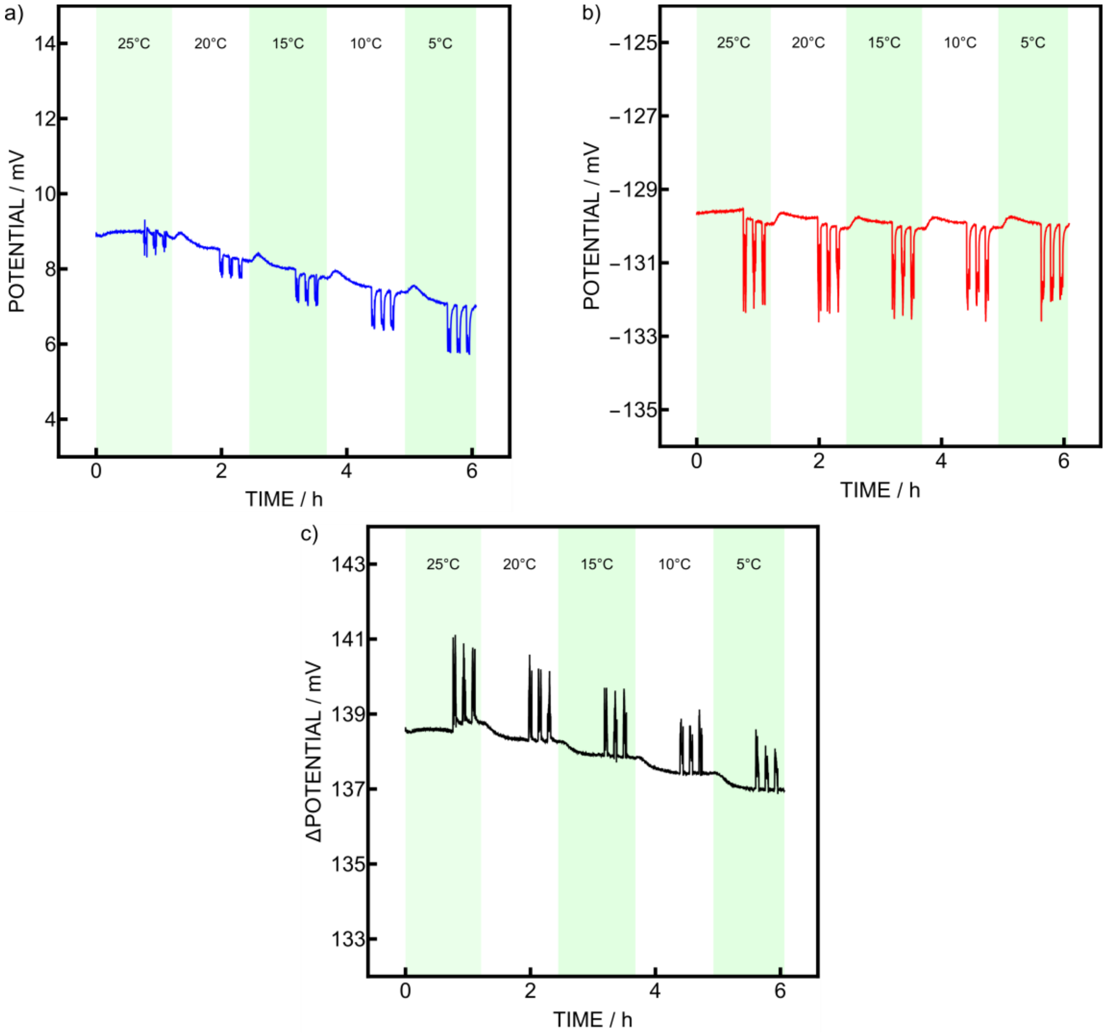

Figure S8. a) Potential trace of sample and b) reference pH glass electrodes against the common reference element in the symmetrical flow cell with NIST phosphate buffer in the sample compartment and NIST borax buffer in the reference compartment at different temperatures. b) Corresponding potentiometric trace of the sample glass electrode measured against the reference glass electrode.
